# Supplementary material for: Competition-driven eco-evolutionary feedback reshapes bacteriophage lambda’s fitness landscape and enables speciation
Source: Nat Commun. 2024 Jan 29;15:863. doi: 10.1038/s41467-024-45008-5 (PMC10825149; doi:10.1038/s41467-024-45008-5)
Supplement: Supplementary file 1 — Supplementary Information [file 41467_2024_45008_MOESM1_ESM.pdf]

Supplementary Figures and Tables for:

Competition-driven eco-evolutionary feedback reshapes bacteriophage lambda's fitness landscape and enables speciation

Michael B. Doud, Animesh Gupta, Victor Li, Sarah J. Medina, Caesar A. De La Fuente, Justin R. Meyer

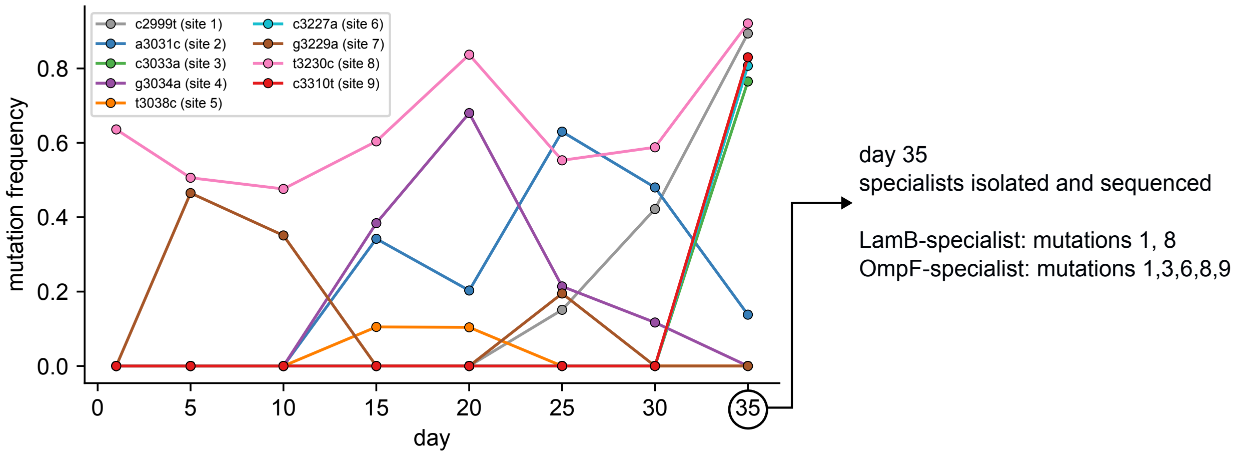

Supplementary Figure 1: **Nine *J* mutations were sampled during λ's evolution from a generalist into two specialists.** Mutations within the *J* gene were detected by population sequencing of λ every 5 days during its laboratory evolution from a generalist (EvoC) into co-existing receptor specialists. At the end of the experiment, two specialist viruses: one LamB-specialist (Specialization Index 0.99986 ± 0.0002 *s.d.*) and one OmpF-specialist (Specialization Index –0.9919 ± 0.0029 *s.d.*) were isolated by plaque purification and the full *J* gene was sequenced for each. The nine mutations observed throughout the course of the laboratory evolution experiment were used as the basis for the design of the combinatorial mutant virus library used to measure fitness landscapes.

sample	total reads	retained reads	total barcodes	retained barcodes	genotypes observed	genotypes observed (%)
lyso_1a	31349993	29995832	10874179	1792161	256/256	**100%**
lyso_1b	29503891	28226421	11013981	1887681	256/256	**100%**
lyso_2a	29720106	28394961	9574206	1600581	256/256	**100%**
lyso_2b	29753717	28424481	9584166	1636865	256/256	**100%**
LibA_pre	21536758	20322420	9615680	1504884	512/512	**100%**
LibA_vGen_1	18485491	17421123	7732259	1286293	362/512	**71%**
LibA_vGen_2	18484112	17349530	7393032	1278832	347/512	**68%**
LibA_vGen_3	18059625	17001858	7353352	1284544	345/512	**67%**
LibA_vGen_LamB_1	18121384	16984788	6304149	1032342	495/512	**97%**
LibA_vGen_LamB_2	20548272	19373665	9263231	1474078	499/512	**97%**
LibA_vGen_LamB_3	28016680	26403612	11034429	1306940	498/512	**97%**
LibA_vGen_OmpF_1	18440873	17399028	7759028	1311059	243/512	**47%**
LibA_vGen_OmpF_2	18308966	17243123	7658868	1262205	242/512	**47%**
LibA_vGen_OmpF_3	21257346	20062589	8431809	1349872	263/512	**51%**
LibA_vLspec_1	18228661	17193555	7117515	1232751	385/512	**75%**
LibA_vLspec_2	22721312	21425682	9218174	1361693	386/512	**75%**
LibA_vLspec_3	19063825	17933074	7432207	1201921	379/512	**74%**
LibA_vOspec_1	21524743	20340163	9150654	1314347	452/512	**88%**
LibA_vOspec_2	21625936	20402090	9174995	1417534	449/512	**88%**
LibA_vOspec_3	19562975	18432341	8910946	1479550	460/512	**90%**
LibB_pre	17314019	16277950	7924903	1375623	512/512	**100%**
LibB_vGen_1	20334575	19169617	9178354	1464099	397/512	**78%**
LibB_vGen_2	21043423	19871734	8334883	1396798	380/512	**74%**
LibB_vGen_3	19619831	18357858	7790406	1189626	373/512	**73%**
LibB_vGen_LamB_1	19519430	18330598	8249694	1368665	451/512	**88%**
LibB_vGen_LamB_2	18545775	17403933	7979985	1186832	441/512	**86%**
LibB_vGen_LamB_3	20855525	19653015	8778775	1360703	437/512	**85%**
LibB_vGen_OmpF_1	18081193	16953548	7952547	1317043	281/512	**55%**
LibB_vGen_OmpF_2	25088144	23644340	10732714	1386740	300/512	**59%**
LibB_vGen_OmpF_3	19434449	18283488	8405639	1418911	287/512	**56%**
LibB_vLspec_1	19713303	18572765	8341297	1368128	421/512	**82%**
LibB_vLspec_2	24433641	23071220	10308241	1508562	426/512	**83%**
LibB_vLspec_3	18427735	17319573	8133495	1400231	423/512	**83%**
LibB_vOspec_1	16508416	15529520	7959310	1331473	463/512	**90%**
LibB_vOspec_2	17624666	16551923	7899799	1353259	460/512	**90%**
LibB_vOspec_3	20110236	18938251	8370929	1369186	467/512	**91%**

Supplementary Table 1: Summary of sequencing read depth and representation of combinatorial genotypes within each sample. Total reads reflects the total number of paired-end sequencing reads; retained reads reflect the number of reads remaining after initial quality control; total barcodes refers to the number of unique molecular barcode sequences observed within each sample; retained barcodes refers to the number of unique molecular barcodes that were observed multiple times in order to correct for sequencing errors by building a consensus genotype sequence per barcode. The number of retained barcodes is the effective sequencing depth. Genotypes observed refers to how many of the 512 combinatorial genotypes were observed in each sample; note that the lysogen library samples (lyso_*) are sub-libraries which, by construction, only contain mutations across 8 of the 9 sites, thus the maximum number of genotypes in each sub-library is 256.

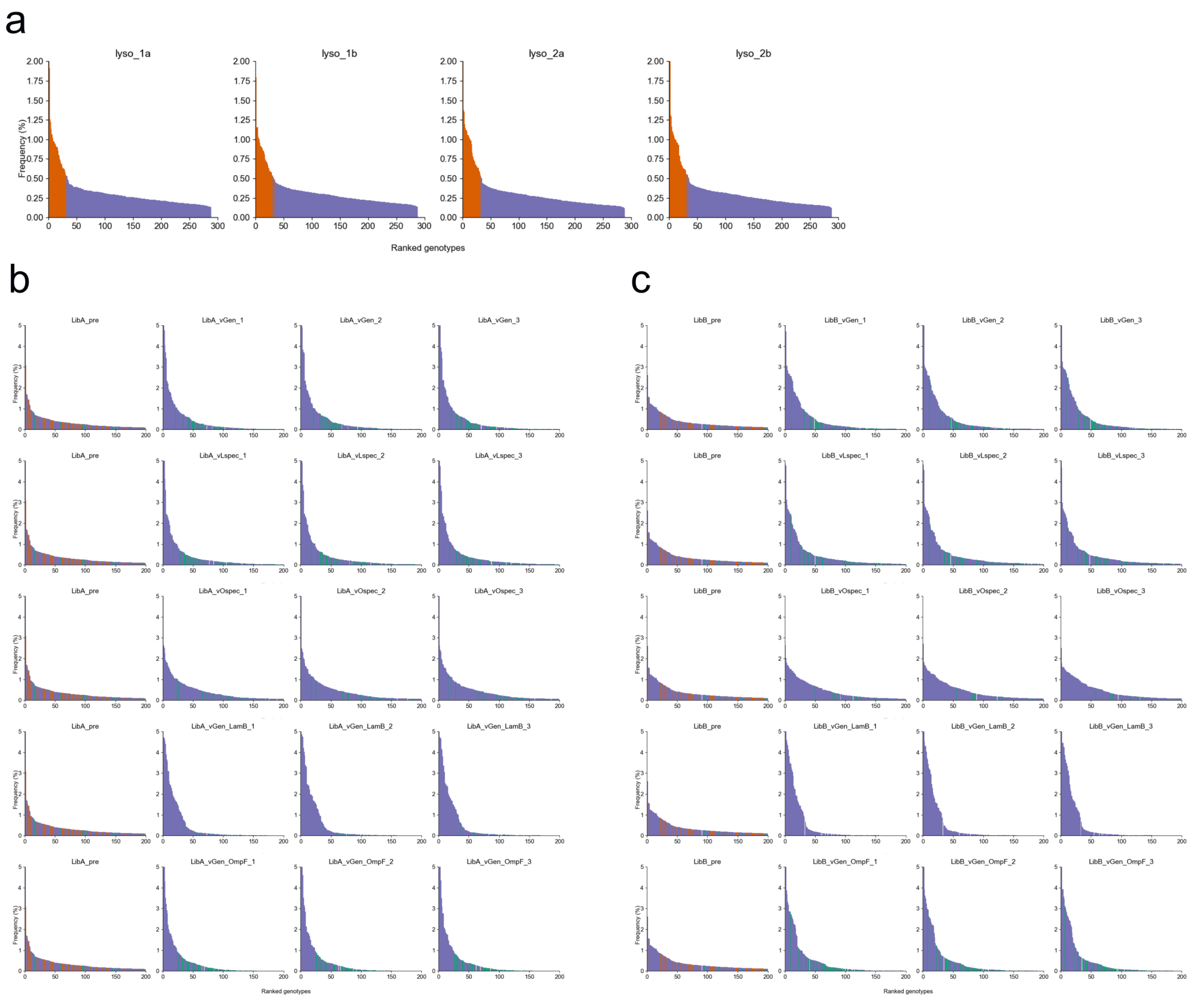

Supplementary Figure 2: Library genotype representation across samples. Genotypes are ordered on the x-axis by frequency rank, and the observed frequency (%) is plotted on the y-axis. Genotypes are colored purple if they are part of the combinatorial library design, red if containing a stop codon in J that was engineered into the pre-mutagenesis sequence for the purposes of purging genotypes that failed to undergo recombination during MAGE, teal if containing the c3283t mutation (note this nucleotide site is not covered in the sequencing amplicon used for the lysogen library samples in panel A), and grey if any other mutation is present in the sequence. **a,** Lysogen sub-libraries sample all 256 intended combinatorial genotypes (purple) at a nearly uniform frequency. Stop-codon containing genotypes (products of incomplete recombination during MAGE) are, as expected, the most frequently observed genotypes. **b, c,** Genotype representation in pre- and post-competition samples for experiments using independently generated virus libraries A (panel B) and B (panel C). Only the 200 most frequent genotypes are plotted for each sample. The pre-competition samples (left-most columns) contain significant numbers of stop-codon containing genotypes (red) due to incomplete purifying selection during the initial induction of viruses from lysogens. After competition experiments (three replicates performed for each condition – rightmost three columns for each panel) there are no longer appreciable levels of stop-codon genotypes, as expected. Some genotypes containing the c3283t mutation (teal) reach appreciable frequencies after competition in some conditions, but the combinatorial library genotypes (purple) dominate in all samples.

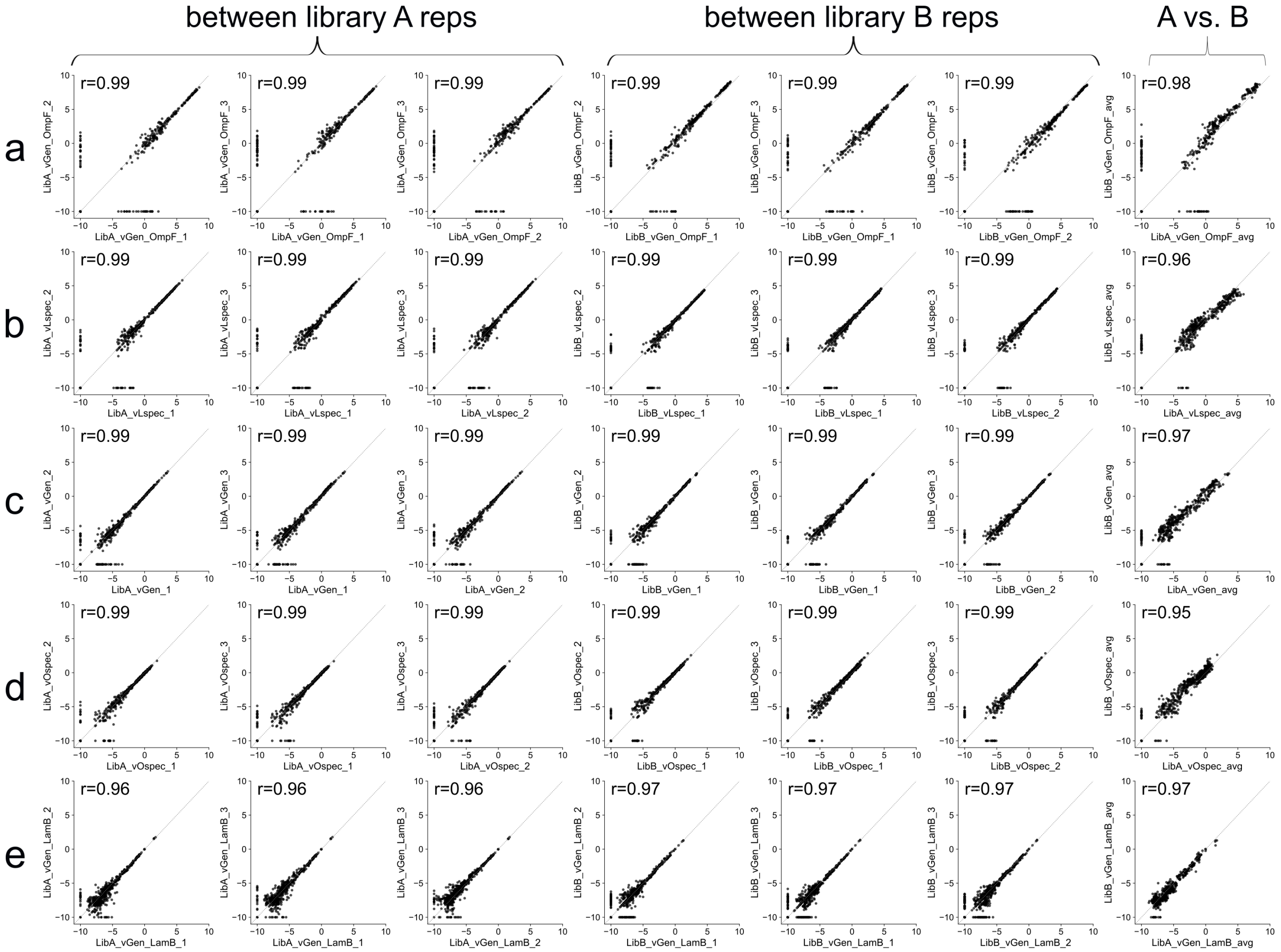

Supplementary Figure 3: Fitness landscape measurements are highly reproducible. Each plot shows the correlation in the measured genotype fitness across the 512 genotypes in the library with the corresponding Pearson's *r* calculated only across the genotypes that were observed in both replicates. Points are plotted at -10 if the genotype was not observed in the corresponding sample. Shown are the correlations across replicate competition flasks using the same library ("between library A reps" and "between library B reps" columns) and between the two libraries ("A vs. B" column). **a,** Competing the library against the generalist with only $OmpF^+$ cells present. **b,** Competing the library against the L-specialist with both cell types. **c,** Competing the library against the generalist with both cell types. **d,** Competing the library against the O-specialist with both cell types. **e,** Competing the library against the generalist with only $LamB^+$ cells present. The environments of competition experiments listed in A-E correspond to those in Fig. 3.

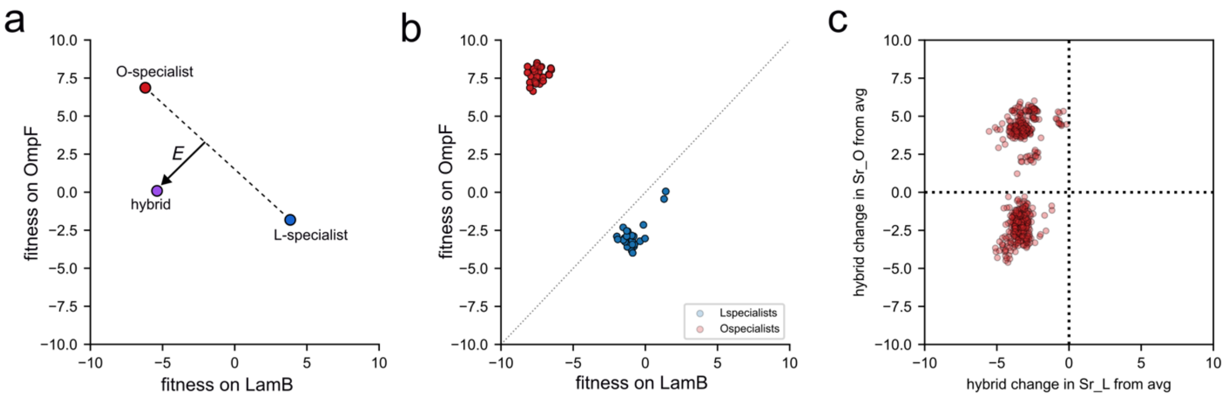

Supplementary Figure 4: Geometric interpretation of fitness effects as a result of hybridization between two specialists. a, Schematic of a theoretical example trio of an O-specialist, an L-specialist, and the resulting hybrid. The dotted line between the two specialists represents an expectation of even compensatory changes in the fitness on one receptor for the other. When the hybrid falls off this line, we compute the distance "*E*" from the line to the hybrid, representing the average change in hybrid fitness from the average of the specialists; in the orientation shown the sign is negative, but if the hybrid is above/to the right of the dotted line the sign is positive. **b,** Fitness of the top 27 specialists of each type used in hybridization analyses. The distribution of "*E*" across all pairwise comparisons of the top 27 specialists of both types is what is plotted in Fig. 2d. **c,** Hybrid genotypes nearly always have loss of fitness on LamB from the parental strains, but the effects of fitness on OmpF are variable.

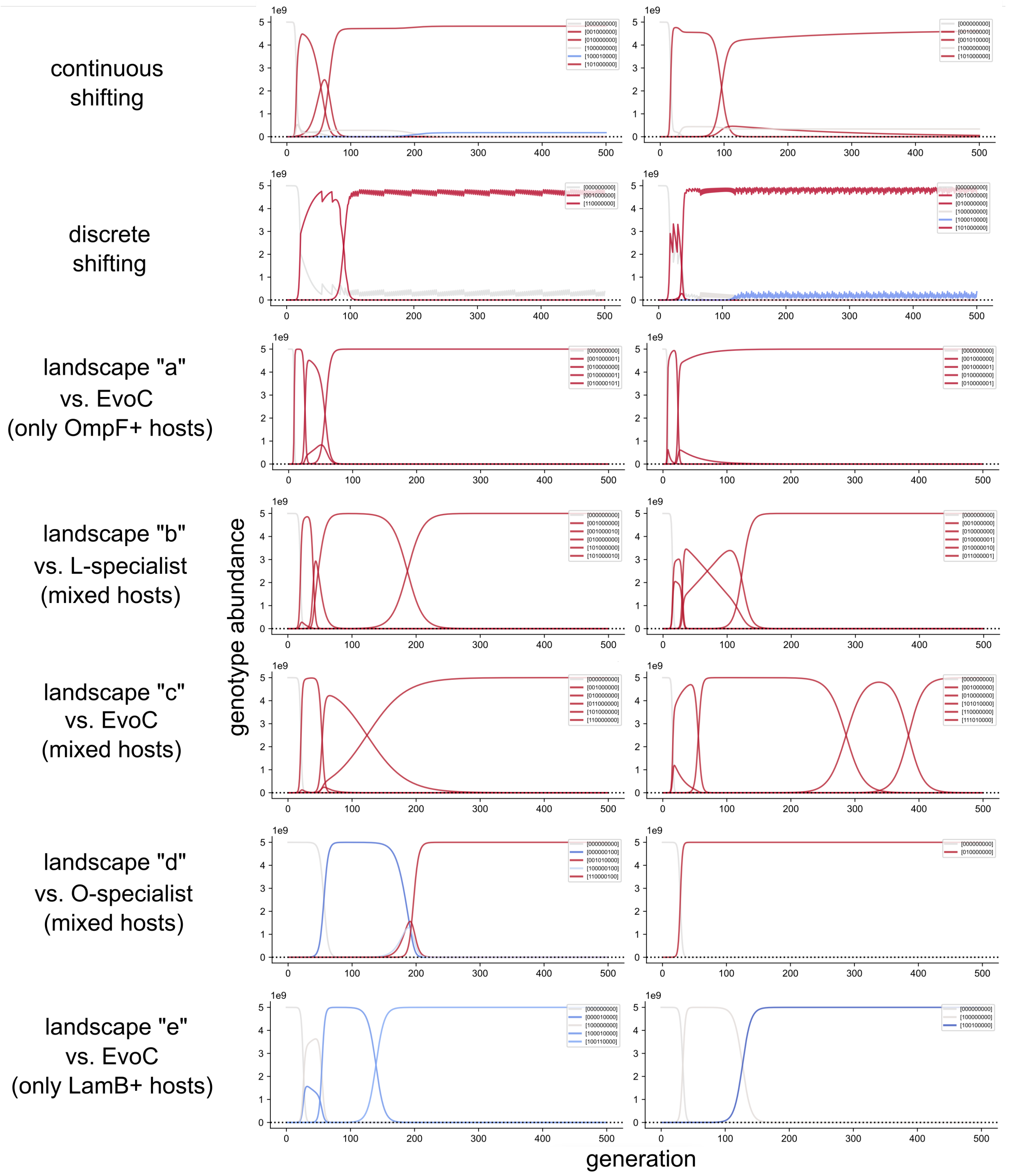

Supplementary Figure 5: Representative population genetic dynamic plots for two simulations under each landscape model. The abundance of each λ genotype is plotted across 500 generations and genotypes are colored by specialization index (as in Fig. 2b; specialization towards OmpF is red and specialization towards LamB is blue). Genotypes are indicated in the legends by a string of nine ‘0’ and ‘1’ characters, representing the ancestral (0) or derived (1) nucleotide at each of the nine mutation sites. Notably, there is genetic heterogeneity maintained under both shifting models; OmpF-specialists dominate in all shifting models, and depending on the simulation either a LamB-specialist or a generalist coexists at a lower frequency. Results of genotypic and phenotypic diversity aggregated across 500 replicate simulations under each model are summarized in Fig. 4. The discrete shifting model produces a characteristic sawtooth-wave like pattern in population abundances as a result of the discrete shifts back and forth between two governing fitness landscapes.

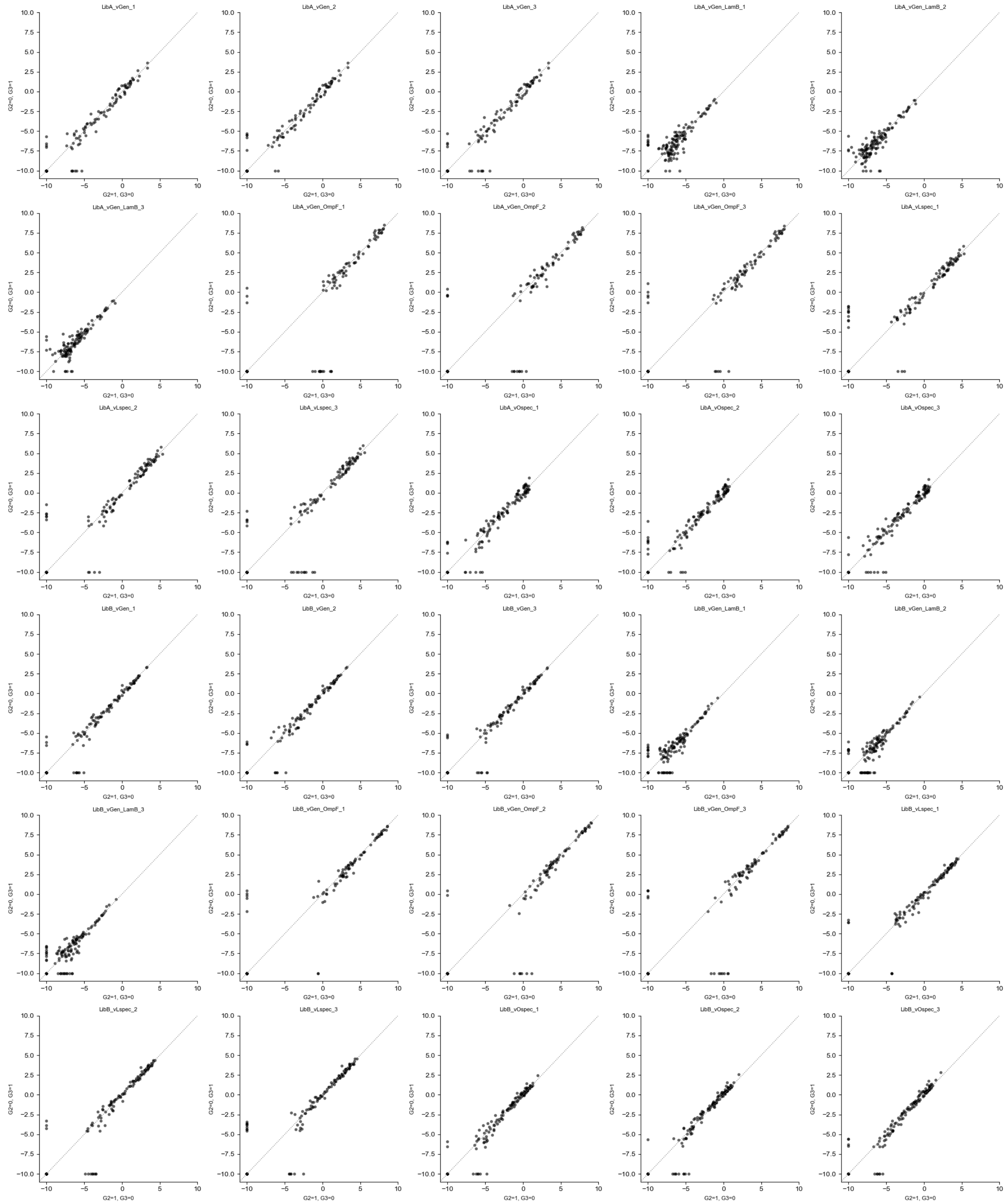

Supplementary Figure 6: Mutations 2 and 3 have similar fitness effects regardless of their genetic background or ecological context. Mutations at sites 2 and 3 are synonymous with each other (each alone, or in combination, produce the same amino-acid change in the protein sequence) and the fitness of genotypes with either mutation 2 or mutation 3 are highly correlated. X-axis: G2=1, G3=0; Y-axis: G2=0, G3=1. Points are plotted at -10 if the genotype was not observed in the corresponding sample. This is similar to Supplementary Figure 7 but shows the fitness effects of the isolated mutation 3 (without mutation 2) on the y-axis.

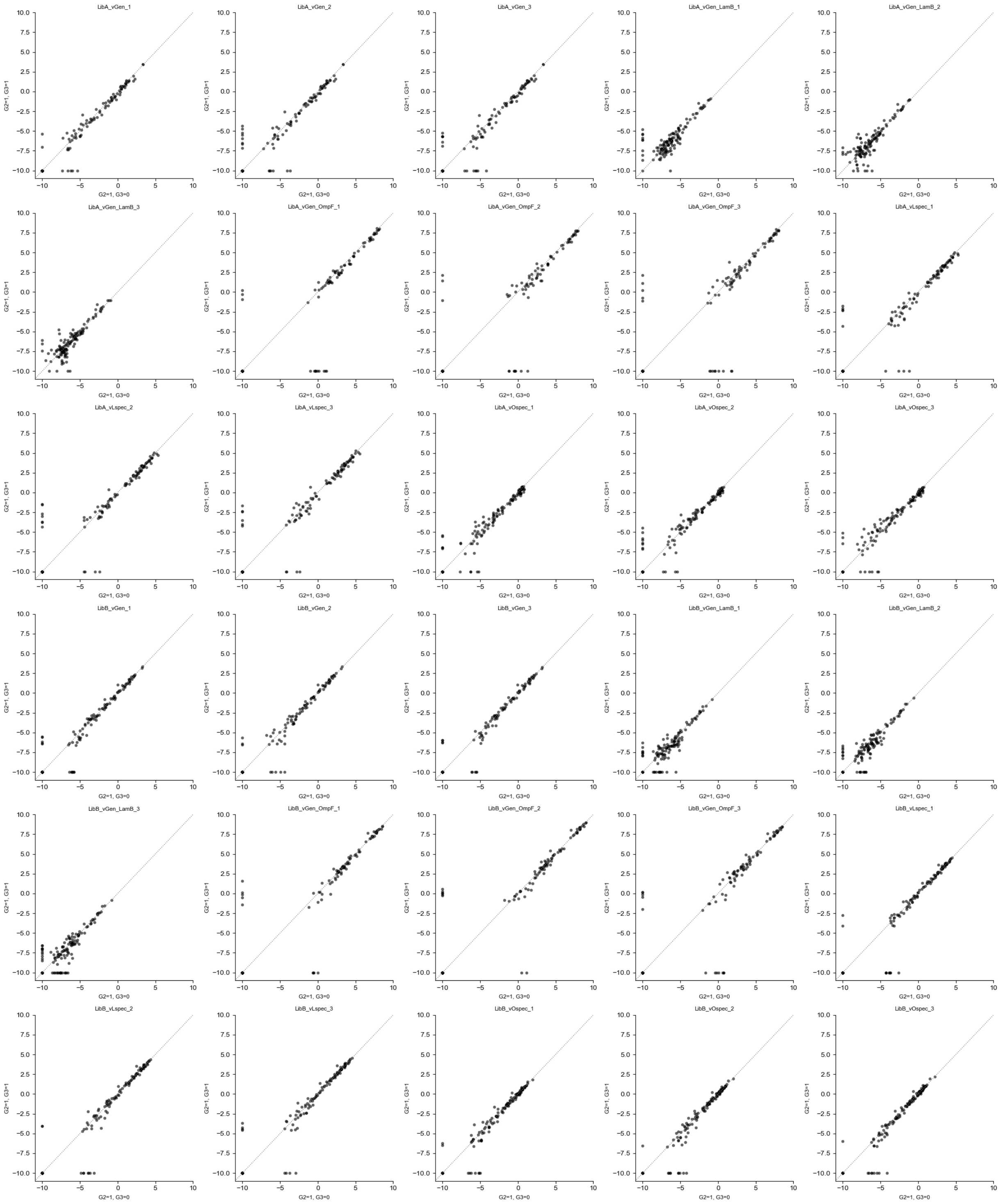

Supplementary Figure 7: Mutations 2 and 2+3 have similar fitness effects regardless of their genetic background or ecological context. Mutations at sites 2 and 3 are synonymous with each other (each alone, or in combination, produce the same amino-acid change in the protein sequence) and the fitness of genotypes with mutation 2 are highly correlated with that of genotypes with the combination of mutations 2 and 3. X-axis: G2=1, G3=0; Y-axis: G2=1, G3=1. Points are plotted at -10 if the genotype was not observed in the corresponding sample. This is similar to Supplementary Figure 6 but shows the fitness effects of the combined mutations 2+3 on the y-axis.

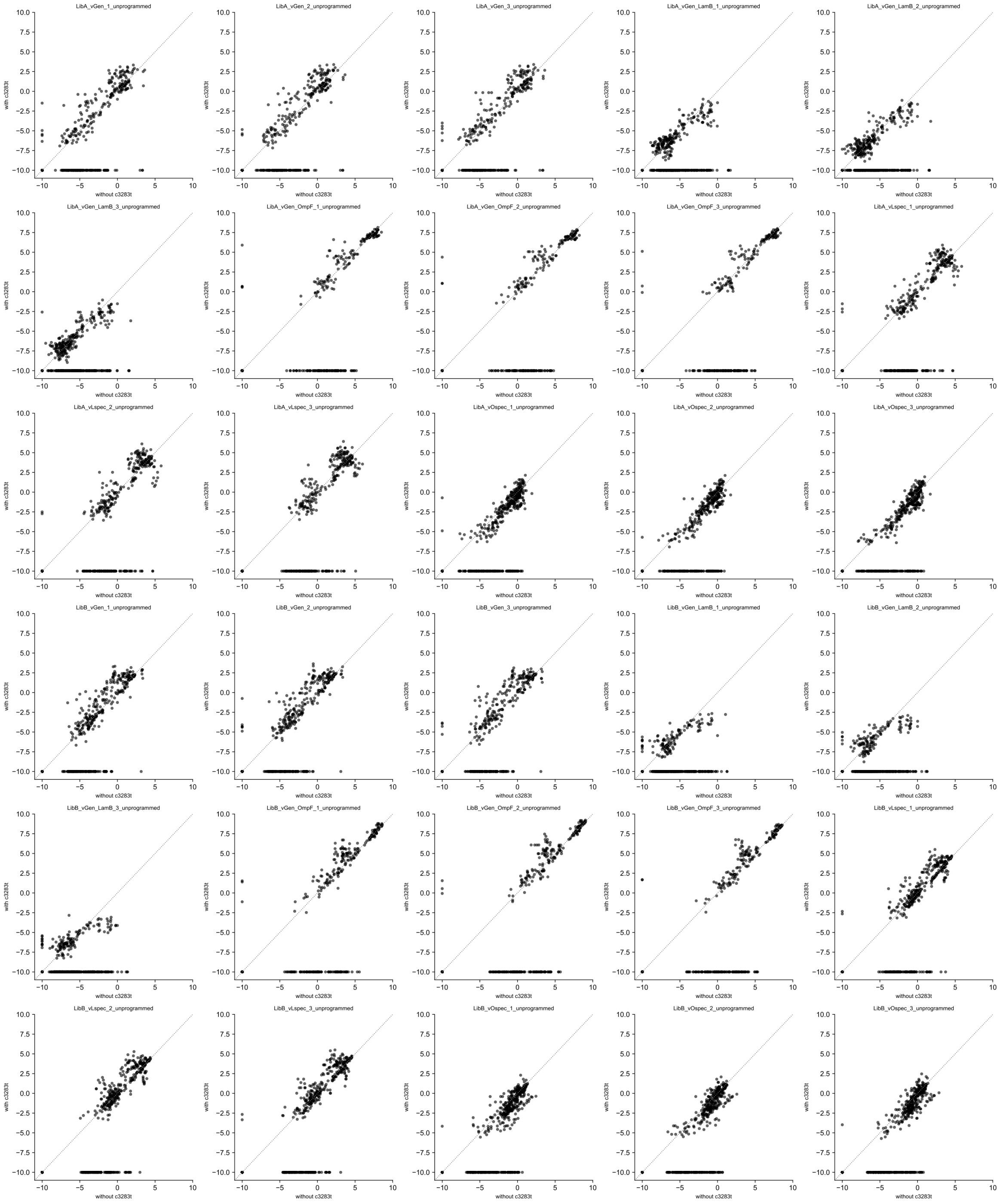

Supplementary Figure 8: Fitness effects of the c3283t mutation. This mutation is not one of the 9 combinatorial mutation sites but was observed at modest frequencies in the pre- and post- competition experiments (Supplementary Figure 2). This mutation was likely present in a minor frequency prior to mutagenesis. Each plot shows the correlation between fitness (selection rate) of a genotype without the c3283t mutation (x-axis) against the fitness of the corresponding genotype with the mutation (y-axis). Points are plotted at -10 if the genotype was not observed in the corresponding sample. Overall, the fitness of genotypes with and without this mutation are moderately correlated, although there appear to be many genotypes that did not have a measured fitness with the mutation (points at y=-10), indicating either strongly deleterious effects of the mutation or lack of representation in the original library construction (we cannot exclude the latter possibility because the sequencing amplicon for the lysogen samples does not cover this nucleotide site). We focus the remaining analysis in this study only on the programmed combinatorial genotypes without extra mutations (c3283t or otherwise).

a

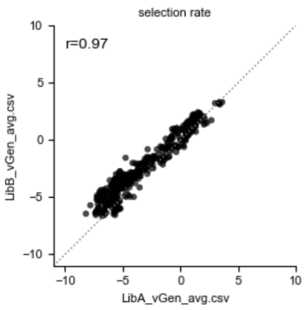

b

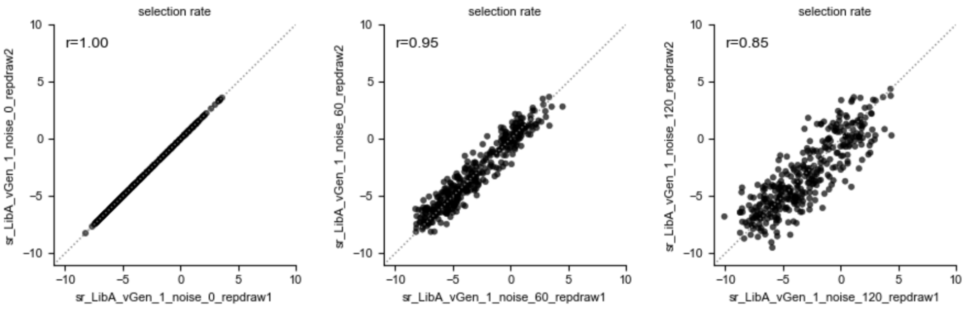

Supplementary Figure 9: Tuning the scale parameter of normally distributed noise added to the fitness landscapes used in replicate simulations of evolution. a, The largest source of experimental noise is between landscapes measured using replicate virus libraries (also see final column of Supplementary Figure 3). **b,** The effect of adding normally distributed noise with scaling parameter of 0, 0.6, and 1.2. The fitness landscapes used to govern evolution in computer simulations were drawn from a distribution of landscapes such that replicate simulations use landscapes as similar to one another as the landscapes measured with biological replicate libraries by selecting a scale parameter (0.6, middle plot) that results in correlations similar to the experiments.

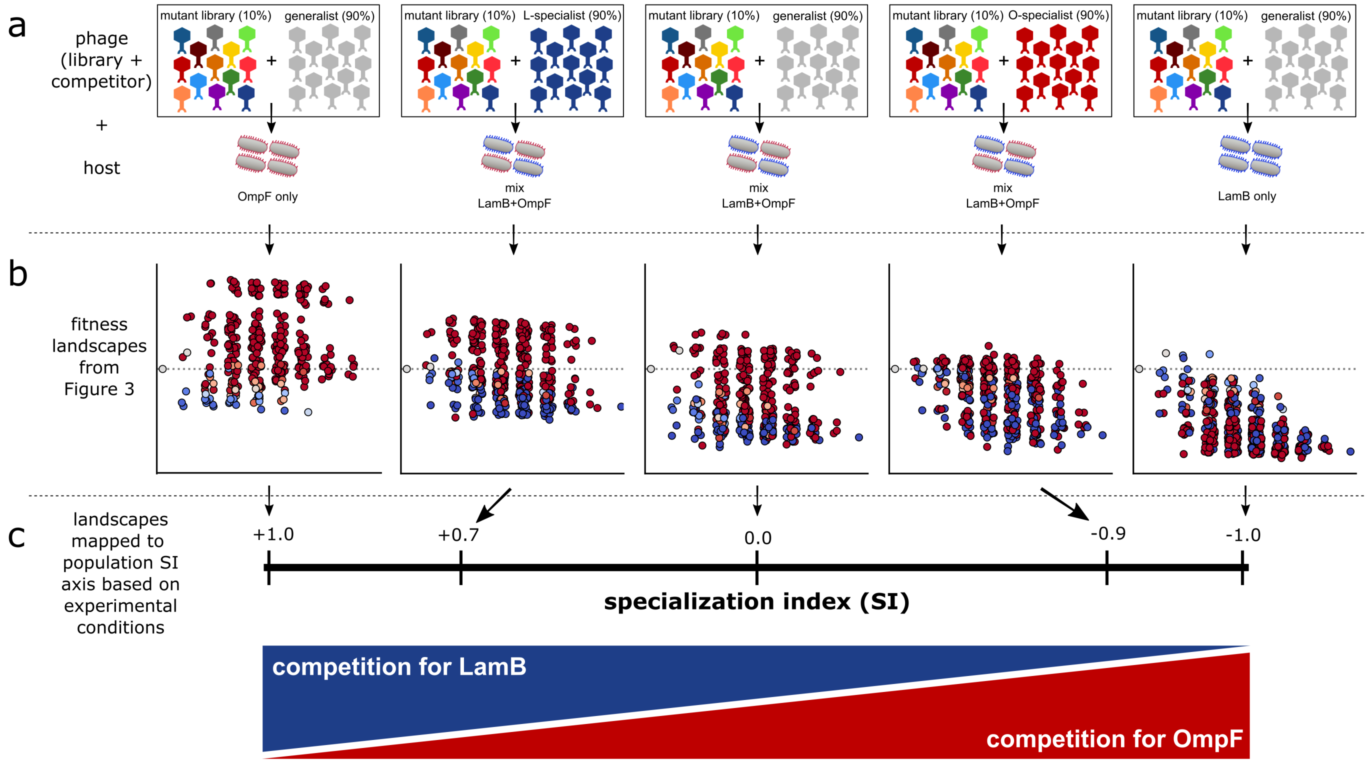

Supplementary Figure 10: Mapping empirical fitness landscapes to a "landscape axis" by population specialization index (SI). a, The experimental conditions that defined each of the five competitive environmental contexts used to measure fitness landscapes; these environments varied by competitor virus and host cell types available for infection. **b**, The five fitness landscapes are reprised from Figure 3. **c**, As described in Methods, landscapes were positioned along a "landscape axis" based on the population SI in the respective experiments; the landscapes measured on a single host cell type were positioned at +1 and -1 since they represent the limiting cases of extreme competition for a receptor such that it is no longer available for infection. The positioning of the landscapes along this axis was used in the shifting models of evolutionary simulations such that the fitness values used to govern selection in each generation of the shifting models were updated either by continuous interpolation between the two adjacent landscapes or by discrete transitions to the nearest empirical landscape as the simulation population SI changed over time. As the population SI shifts to the left (representing more abundant L-specialists), competition for LamB intensifies, resulting in higher fitness values for OmpF-specialists, and vice-versa. In the *continuous shifting landscape simulation models*, the SI of the simulated population is used to position the population on the SI axis; if the population SI is equal to the SI of an empirical fitness landscape from Figure 3 then that landscape is used to govern selection during that generation, but most of the time the simulated population SI lies somewhere between two adjacent empirical landscapes and a linear interpolation of fitness values between the two empirical landscapes is used to determine fitness values to govern selection. In the *discrete shifting landscape simulation models*, the empirical landscape with the SI nearest to the simulation population SI is used.

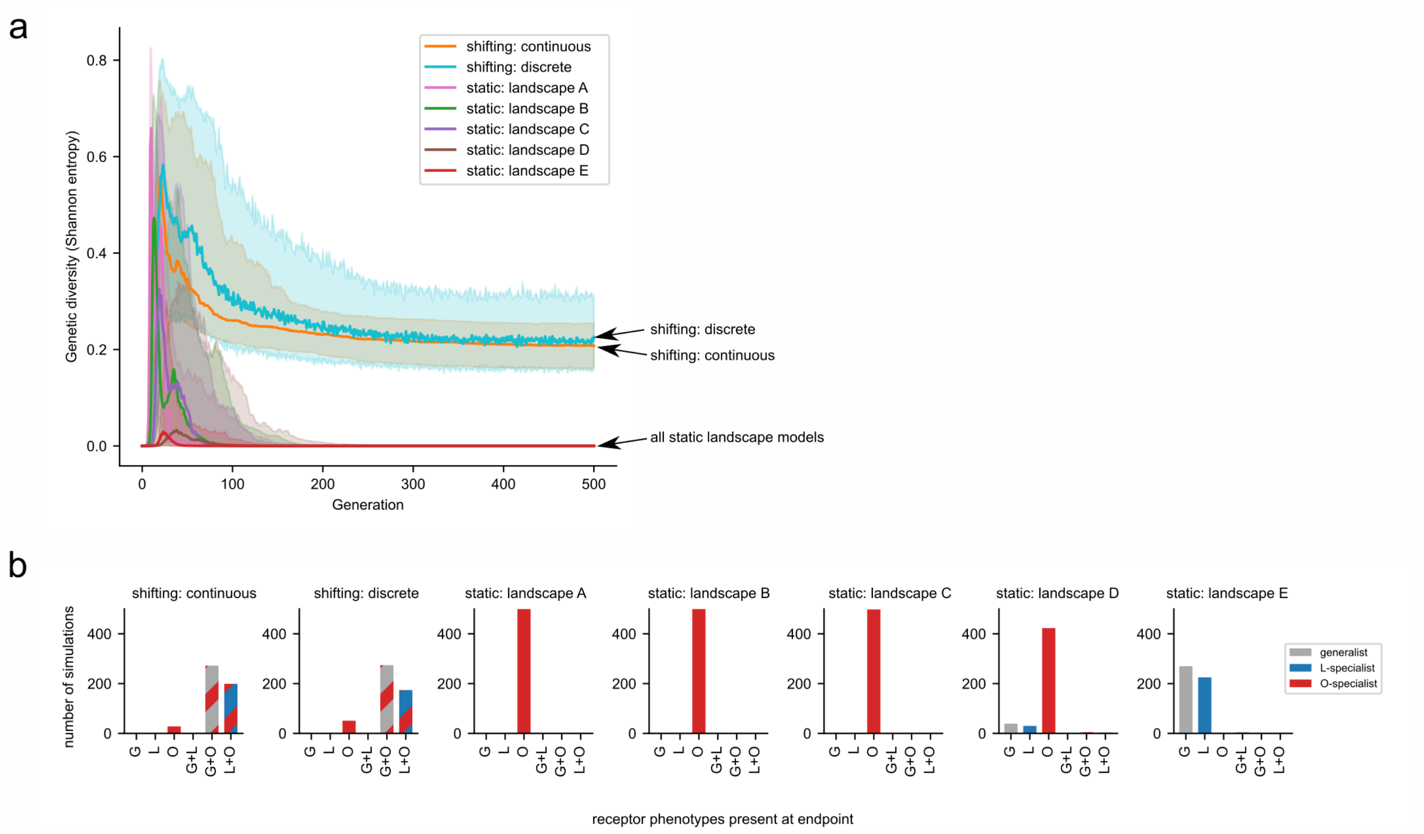

Supplementary Figure 11: Both continuous and discrete shifting landscape models result in similar genetic and phenotypic diversity. The same analysis of genetic and phenotypic diversity as presented in Fig. 4 is shown here, but in this figure the analysis includes the discrete shifting model. **a,** Genetic diversity computed at each generation; solid lines represent the median and shaded regions represent the interquartile range. The endpoint diversity for the models are labeled with arrows for clarity. **b,** Phenotypic diversity computed at the endpoint of each simulation. The number of simulations arriving at the designated combination of phenotypes (>= 2.5% abundance) is plotted.

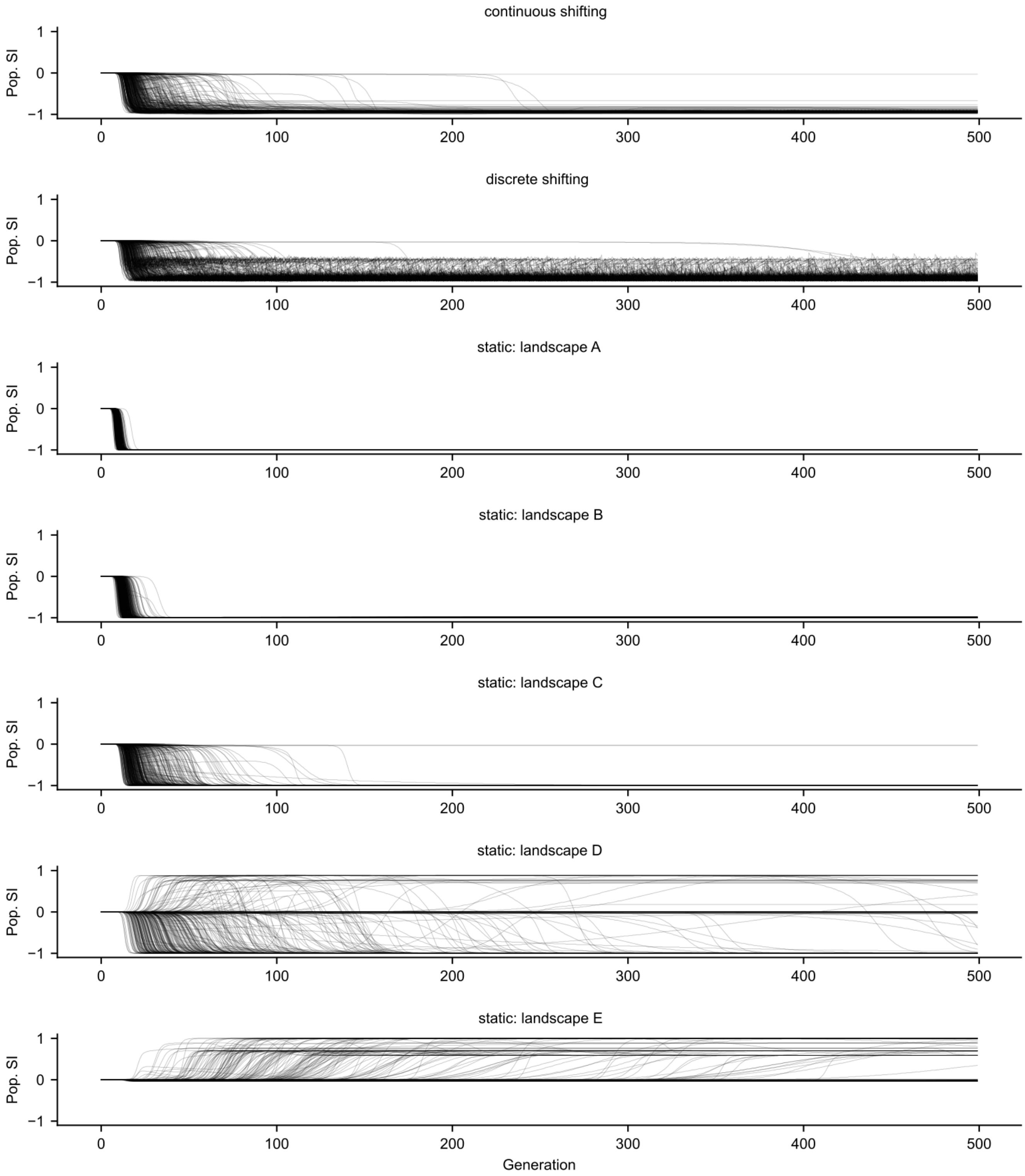

Supplementary Figure 12: Trajectories of simulated population specialization index for all replicate simulations under each landscape model. The population SI trajectory for each simulation is plotted as a semi-transparent line, for all 500 replicate simulations under each landscape model. The shifting landscape models tend to reach an equilibrium around a population SI between -0.9 and -1.0, in contrast to the static models which tend to reach equilibrium SI near +/- 1.0 as they become fixed with single specialist genotypes and phenotypes (as seen in Fig. 4; also see Supplementary Fig. 13 for trajectories of the median SI across simulations within each model). Landscapes A, B, and C all reach an endpoint with low genetic diversity dominated by O-specialists (as seen in Fig. 4, Supplementary Fig. 11), but the time it takes to reach this equilibrium is shortest in the most extreme competitive environment (landscape A, characterized by the highest fitness peaks of the dominating O-specialists). There is more stochasticity across simulations in static landscapes D and E, which is also reflected in the different specialist endpoints reached as shown in Fig. 4b. Landscape E is unique in that the fitness peaks are defined by both generalists and L-specialists (see Fig. 3e); the evolutionary trajectories of SI most often equilibrate to a very slightly negative SI value corresponding to a dominating generalist, but less often also equilibrate to a relatively positive SI corresponding to a dominating L-specialist.

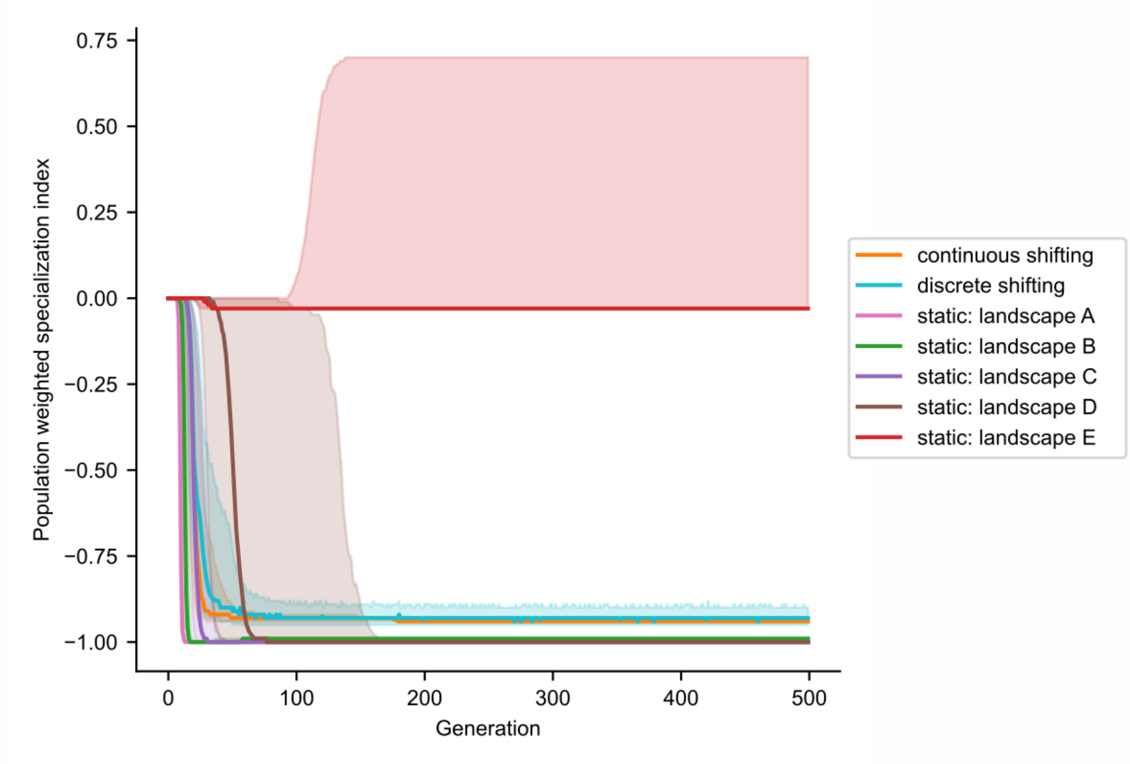

Supplementary Figure 13: Median and interquartile range of trajectories of simulated population specialization index under each landscape model. The median SI is shown as a bold line and the shaded region shows the interquartile range. This figure summarizes the individual simulation data trajectories shown in Supplementary Fig. 12.

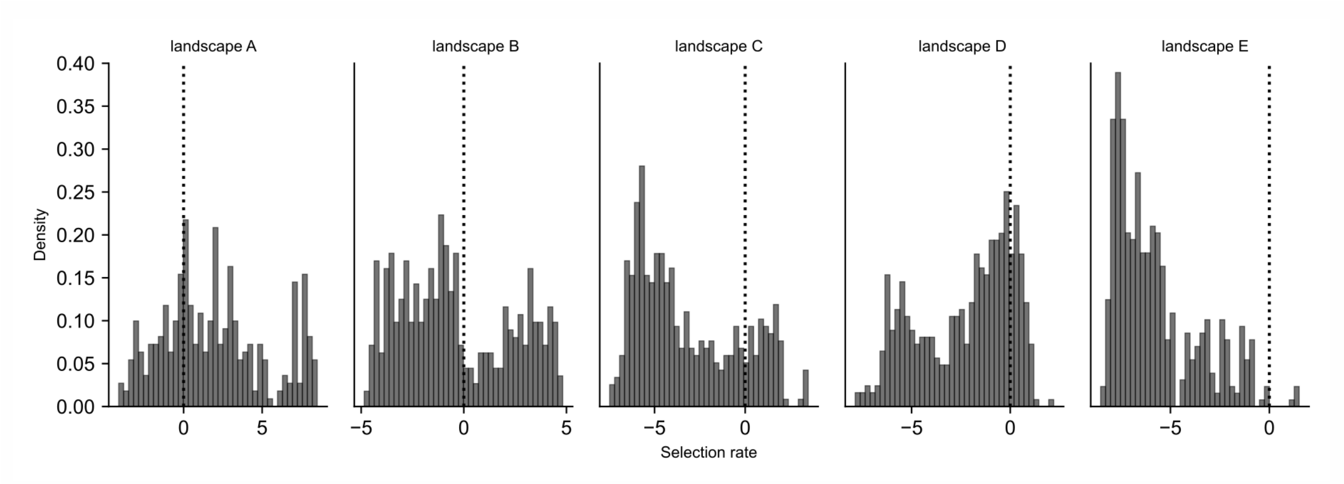

Supplementary Figure 14: Histograms showing the distribution of fitness effects (DFE) for each landscape. Each histogram shows the density of genotypes with the specified selection rates for the fitness landscapes as defined in Fig. 3.

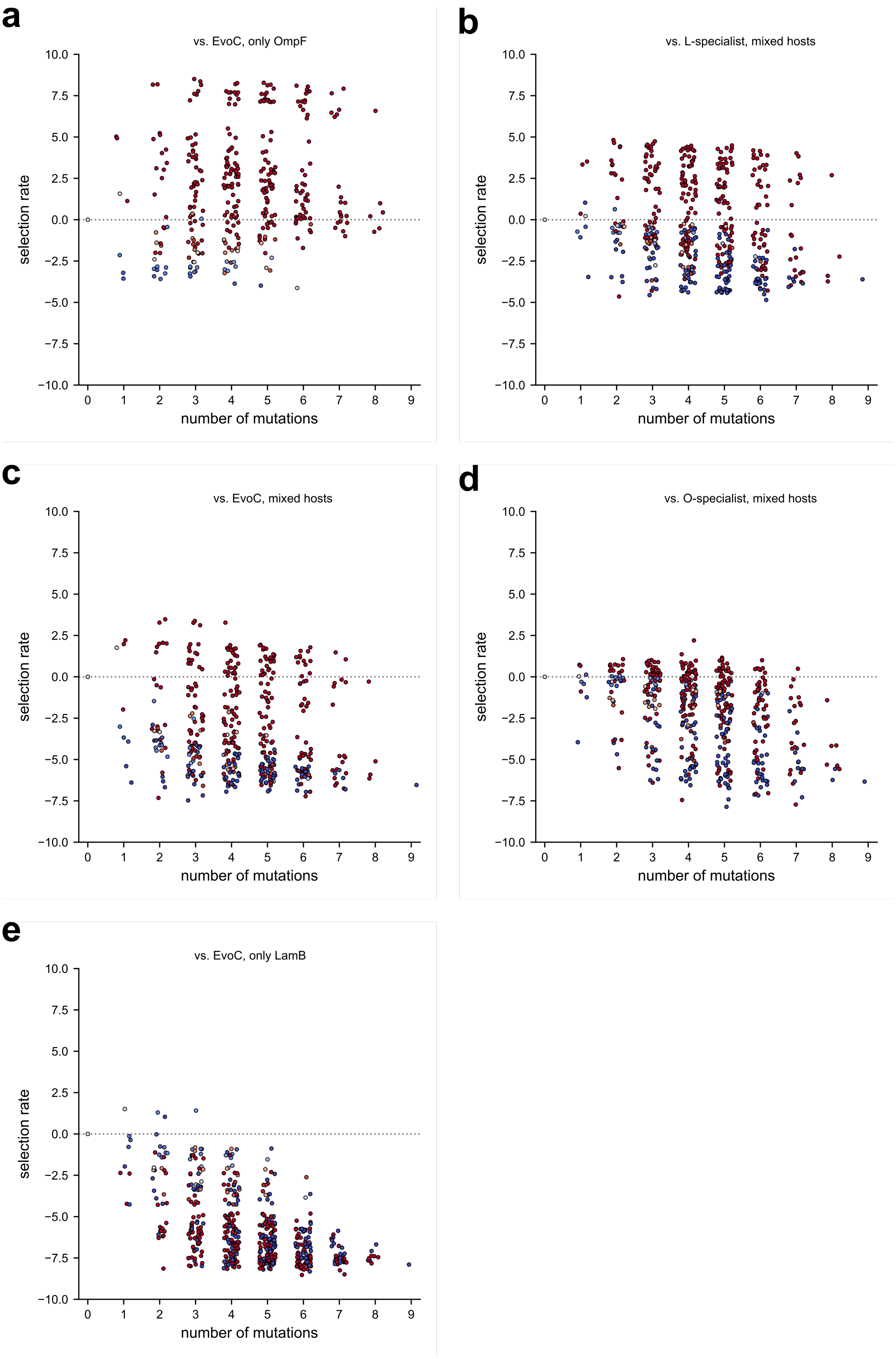

Supplementary Figure 15: Fitness landscapes as presented in Fig. 3 with modifications for clarity. Compared to Fig. 3, these plots use smaller points and no lines between neighboring genotypes, for the purposes of better visualizing individual points. Points are colored by specialization index as defined in Figure 2b.
