# Supplementary material for: Competition-driven eco-evolutionary feedback reshapes bacteriophage lambda’s fitness landscape and enables speciation
Source: Nat Commun. 2024 Jan 29;15:863. doi: 10.1038/s41467-024-45008-5 (PMC10825149; doi:10.1038/s41467-024-45008-5)
Supplement: Supplementary file 3 — Reporting Summary [file 41467_2024_45008_MOESM3_ESM.pdf]

nature portfolio

Corresponding author(s): Justin Meyer

Last updated by author(s): Jan 4, 2024

# Reporting Summary

Nature Portfolio wishes to improve the reproducibility of the work that we publish. This form provides structure for consistency and transparency in reporting. For further information on Nature Portfolio policies, see our Editorial Policies and the Editorial Policy Checklist.

## Statistics

For all statistical analyses, confirm that the following items are present in the figure legend, table legend, main text, or Methods section.

| n/a | Confirmed | |
|---|---|---|
| ☐ | ☒ | The exact sample size (*n*) for each experimental group/condition, given as a discrete number and unit of measurement |
| ☐ | ☒ | A statement on whether measurements were taken from distinct samples or whether the same sample was measured repeatedly |
| ☒ | ☐ | The statistical test(s) used AND whether they are one- or two-sided<br>*Only common tests should be described solely by name; describe more complex techniques in the Methods section.* |
| ☒ | ☐ | A description of all covariates tested |
| ☒ | ☐ | A description of any assumptions or corrections, such as tests of normality and adjustment for multiple comparisons |
| ☐ | ☒ | A full description of the statistical parameters including central tendency (e.g. means) or other basic estimates (e.g. regression coefficient) AND variation (e.g. standard deviation) or associated estimates of uncertainty (e.g. confidence intervals) |
| ☒ | ☐ | For null hypothesis testing, the test statistic (e.g. *F*, *t*, *r*) with confidence intervals, effect sizes, degrees of freedom and *P* value noted<br>*Give P values as exact values whenever suitable.* |
| ☒ | ☐ | For Bayesian analysis, information on the choice of priors and Markov chain Monte Carlo settings |
| ☒ | ☐ | For hierarchical and complex designs, identification of the appropriate level for tests and full reporting of outcomes |
| ☐ | ☒ | Estimates of effect sizes (e.g. Cohen's *d*, Pearson's *r*), indicating how they were calculated |

*Our web collection on statistics for biologists contains articles on many of the points above.*

## Software and code

Policy information about availability of computer code

| | |
|---|---|
| Data collection | No software was used for data collection |
| Data analysis | The computer code for computational analyses and figure generation is available in a GitHub repository at github.com/mbdoud/shifting-fitness-landscapes and has been deposited at Zenodo (DOI: 10.5281/zenodo.10459906). The computational analyses were run using python 3.11.4, numpy 1.25.1, scipy 1.11.1, scikit-learn 1.3.0, pandas 2.0.3, networkx 3.1, matplotlib 3.7.2, and pysam 0.21.0. |

For manuscripts utilizing custom algorithms or software that are central to the research but not yet described in published literature, software must be made available to editors and reviewers. We strongly encourage code deposition in a community repository (e.g. GitHub). See the Nature Portfolio guidelines for submitting code & software for further information.

## Data

Policy information about availability of data

All manuscripts must include a data availability statement. This statement should provide the following information, where applicable:

- Accession codes, unique identifiers, or web links for publicly available datasets
- A description of any restrictions on data availability
- For clinical datasets or third party data, please ensure that the statement adheres to our policy

Sequencing data are available from the Sequence Read Archive under BioProject PRJNA984687, BioSample Accession SAMN35774826 (https://

www.ncbi.nlm.nih.gov/sra/?term=SAMN35774826). All data analysis intermediate datasets downstream from sequencing data, including the simulation datasets, are deposited in the GitHub repository and archived at Zenodo (DOI: 10.5281/zenodo.10459906).

## Research involving human participants, their data, or biological material

Policy information about studies with human participants or human data. See also policy information about sex, gender (identity/presentation), and sexual orientation and race, ethnicity and racism.

| | |
|---|---|
| Reporting on sex and gender | N/A |
| Reporting on race, ethnicity, or other socially relevant groupings | N/A |
| Population characteristics | N/A |
| Recruitment | N/A |
| Ethics oversight | N/A |

Note that full information on the approval of the study protocol must also be provided in the manuscript.

# Field-specific reporting

Please select the one below that is the best fit for your research. If you are not sure, read the appropriate sections before making your selection.

☒ Life sciences ☐ Behavioural & social sciences ☐ Ecological, evolutionary & environmental sciences

For a reference copy of the document with all sections, see nature.com/documents/nr-reporting-summary-flat.pdf

# Life sciences study design

All studies must disclose on these points even when the disclosure is negative.

| | |
|---|---|
| Sample size | No sample-size calculations were performed. Sample size for the mutant virus library used to measure the fitness landscapes was chosen to be one million plaque-forming units in order to thoroughly sample the library complexity of 512 unique variants, and replication studies (Supplementary Figure 3) demonstrated this sample size was sufficiently large to avoid stochastic bottlenecking of library variants. Fitness landscapes were computed based on changes in mutant virus genotype frequency. Genotype frequencies were computed with deep sequencing at depths far exceeding the complexity of the libraries (Supplementary Table 1). The sample size for number of replicate simulations was arbitrarily chosen to be 500, which is sufficient to capture a range of stochastic behavior within the simulations (Figure 4b, Supplementary Figure 11, Supplementary Figure 12). |
| Data exclusions | No data were excluded from the analyses. |
| Replication | Two independently-generated mutant virus libraries were used. Every experimental condition for the measurement of fitness landscapes was fully repeated in experimental triplicate. There were high levels of reproducibility between virus libraries and between triplciate experiments of each library (Figure 1D, Supplementary Figure 3) |
| Randomization | Randomization is not relevant to the experimental design because there were no covariates to control for in the experimental design. |
| Blinding | Blinding was not relevant to the study because there were no aspects of the study design susceptible to subjective bias. |

# Reporting for specific materials, systems and methods

We require information from authors about some types of materials, experimental systems and methods used in many studies. Here, indicate whether each material, system or method listed is relevant to your study. If you are not sure if a list item applies to your research, read the appropriate section before selecting a response.

## Materials & experimental systems

| n/a | Involved in the study |
| --- | --- |
| ☒ | ☐ Antibodies |
| ☒ | ☐ Eukaryotic cell lines |
| ☒ | ☐ Palaeontology and archaeology |
| ☐ | ☒ Animals and other organisms |
| ☒ | ☐ Clinical data |
| ☒ | ☐ Dual use research of concern |
| ☒ | ☐ Plants |

## Methods

| n/a | Involved in the study |
| --- | --- |
| ☒ | ☐ ChIP-seq |
| ☒ | ☐ Flow cytometry |
| ☒ | ☐ MRI-based neuroimaging |

# Animals and other research organisms

Policy information about studies involving animals; ARRIVE guidelines recommended for reporting animal research, and Sex and Gender in Research

| | |
| --- | --- |
| Laboratory animals | No animals were used.<br><br>Laboratory strains of Escherichia coli were used: Bacterial hosts used for evolution and competition experiments were the LamB ("L–" strain JW3996) and OmpF ("O–" strain JW0912) knockout strains from the Keio collection. The "wild type" E. coli K-12 BW25113 (parental strain in the Keio collection) was used unless otherwise stated to titer λ phages by plaque assay. The host strain HWEC106, which contains a mutation in the mutS mismatch repair gene and the pKD46 plasmid containing the arabinose-inducible λ red recombineering machinery, was used to perform mutagenesis and is the strain that we used to maintain the cI26-derived engineered lysogen library.<br><br>Speciation replay experiments used strictly lytic phages derived from the λ phage strain cI26. We engineered a lysogenic form of cI26 by replacing the nonfunctional cI gene with a temperature-sensitive mutant (to enable heat induction of the lytic pathway) and adding a constitutively expressed chloramphenicol resistance gene for antibiotic selection of lysogens in the bacterial host HWEC106. |
| Wild animals | No wild animals were used. |
| Reporting on sex | N/A. |
| Field-collected samples | N/A. |
| Ethics oversight | All work was performed under the UC San Diego Biological Use Agreement to ensure safe and ethical practices. |

Note that full information on the approval of the study protocol must also be provided in the manuscript.
